# Supplementary material for: Imp and Chinmo are required for embryonic motor neuron axon and dendrite targeting
Source: Biol Open. 2025 Jul 25;14(7):bio062105. doi: 10.1242/bio.062105 (PMC12352280; doi:10.1242/bio.062105)
Supplement: Supplementary information [file biolopen-14-062105-s1.pdf]

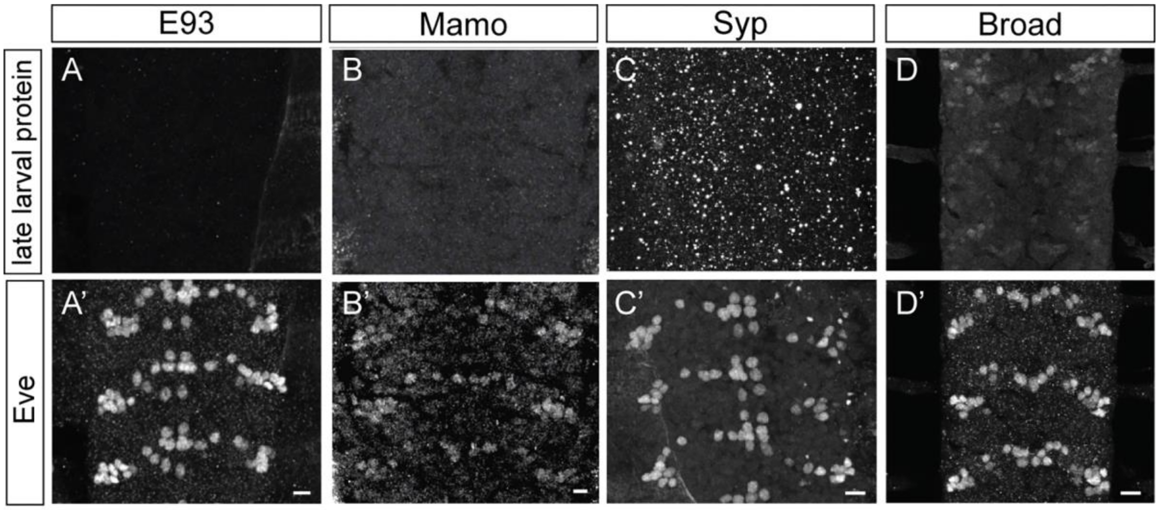

Supplemental Figure 1

Fig. S1. Late larval proteins show little or no expression in embryos.

E93 (A, A'), Mamo (B, B'), and Syp (C, C'), are not expressed in the embryonic VNC. Broad (D, D') is expressed in a subset of cells. Eve is shown as a fiduciary marker for a subset of post-mitotic motoneurons and interneurons. Stage 17 shown; anterior up; scale bar 5μm.

Biology Open • Supplementary information

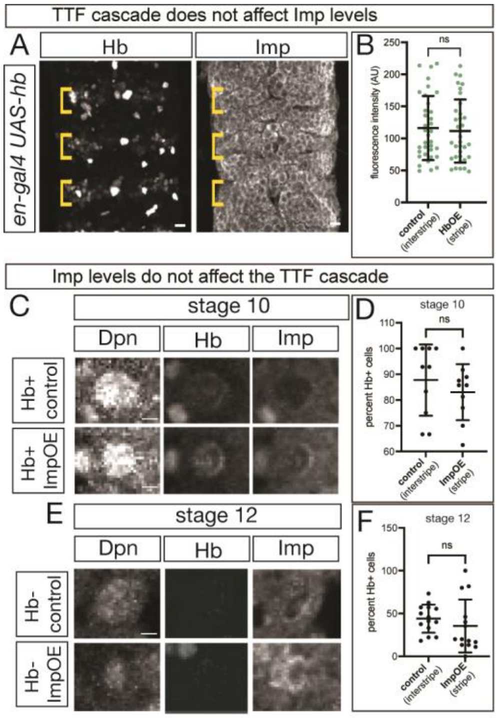

Fig. S2. The embryonic TTF cascade does not affect Imp levels

(A-B) Overexpression of Hb does not alter Imp expression. (A) Hb is overexpressed in a stripe pattern via *en-gal4 UAS-hb*. Control, interstripe domain; Hb overexpression, stripe domain (brackets). (B) Quantification. n = 9 embryos. Ventral view. Scale bar, 5 µm.

(C-F) Overexpression of Imp does not alter Hb expression in NBs. A) Imp is overexpressed in a stripe pattern via *en-gal4 UAS-Imp*. Control, interstripe domain; Hb overexpression, stripe domain. C) The majority of Stage 10 NBs express Hb, which is consistent in ImpOE stripes. Scale bar, 2 µm. D) quantitation of NBs expressing Hb n > 100 NBs in at least 3 embryos per stage (p>0.05, ns, unpaired t-test).

(E) The majority of Stage 12 NBs express Hb, which is consistent in ImpOE stripes. Scale bar, 2 µm. (F) Quantitation of NBs expressing Hb n > 100 NBs in at least 3 embryos per stage (p>0.05, ns, unpaired t-test).

Biology Open • Supplementary information

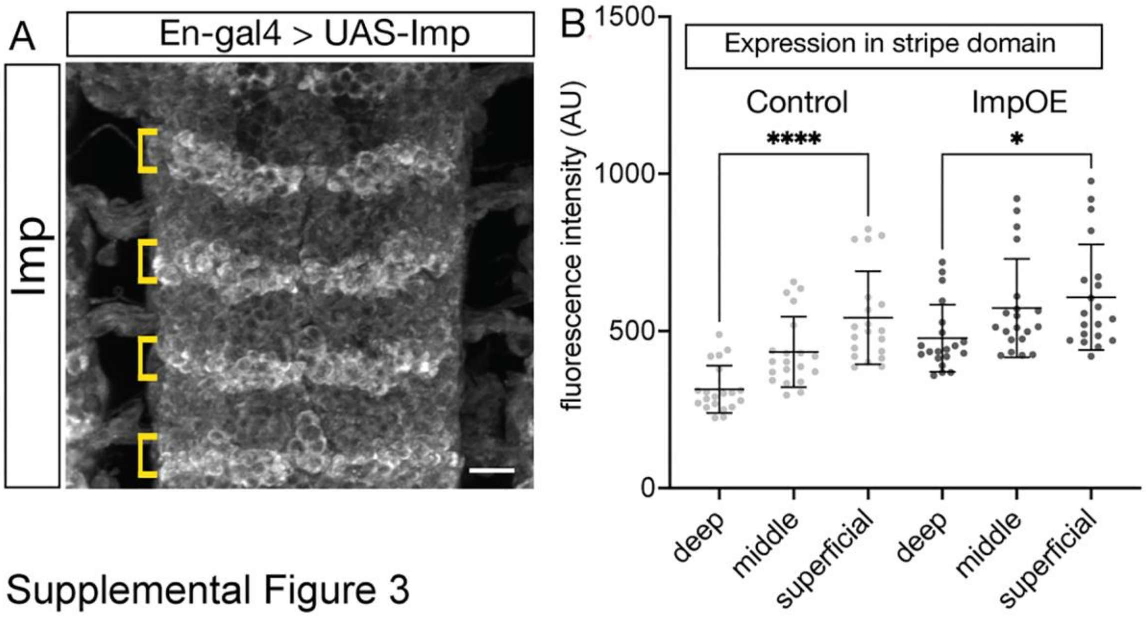

Fig. S3. Imp overexpression can flatten the Imp temporal gradient.

(A) Imp overexpression in stripes (brackets) via *en-gal4 UAS-Imp* transgene. Ventral view, anterior up, scale bar, 5μM.

(B) Imp overexpression leads to a flattening of the Imp gradient within the stripe domain. Control, interstripe domain; Imp overexpression, stripe domain. Quantitation. (control: $p>0.05$, deep vs superficial $p>0.0001$; ImpOE: $p>0.05$, deep vs superficial $p >0.05$, One-way ANOVA analysis with Tukey's multiple comparisons test).
